# A Discussion of Virtual Reality As a New Tool for Training Healthcare Professionals

**DOI:** 10.3389/fpubh.2018.00044

**Published:** 2018-02-26

**Authors:** Caroline Fertleman, Phoebe Aubugeau-Williams, Carmel Sher, Ai-Nee Lim, Sophie Lumley, Sylvie Delacroix, Xueni Pan

**Affiliations:** ^1^UCL Medical School, London, United Kingdom; ^2^Whittington Hospital, London, United Kingdom; ^3^The Heron Practice, London, United Kingdom; ^4^Department of Microbiology, Whittington Hospital, London, United Kingdom; ^5^National Medical Director’s Clinical Fellow Scheme, Faculty of Medical Leadership and Management, London, United Kingdom; ^6^Faculty of Laws, University College London, London, United Kingdom; ^7^Department of Computer Science, Goldsmiths University of London, London, United Kingdom

**Keywords:** virtual reality, medical education, antibiotic stewardship, communication skills, antibacterial resistance

## Abstract

**Background:**

Virtual reality technology is an exciting and emerging field with vast applications. Our study sets out the viewpoint that virtual reality software could be a new focus of direction in the development of training tools in medical education. We carried out a panel discussion at the Center for Behavior Change 3rd Annual Conference, prompted by the study, “The Responses of Medical General Practitioners to Unreasonable Patient Demand for Antibiotics––A Study of Medical Ethics Using Immersive Virtual Reality” (1).

**Methods:**

In Pan et al.’s study, 21 general practitioners (GPs) and GP trainees took part in a videoed, 15-min virtual reality scenario involving unnecessary patient demands for antibiotics. This paper was discussed in-depth at the Center for Behavior Change 3rd Annual Conference; the content of this paper is a culmination of findings and feedback from the panel discussion. The experts involved have backgrounds in virtual reality, general practice, medicines management, medical education and training, ethics, and philosophy.

**Viewpoint:**

Virtual reality is an unexplored methodology to instigate positive behavioral change among clinicians where other methods have been unsuccessful, such as antimicrobial stewardship. There are several arguments in favor of use of virtual reality in medical education: it can be used for “difficult to simulate” scenarios and to standardize a scenario, for example, for use in exams. However, there are limitations to its usefulness because of the cost implications and the lack of evidence that it results in demonstrable behavior change.

## Background

Virtual reality technology describes the use of headsets displaying a particular environment to simulate a user’s physical existence in a virtual or imaginary setting. Headsets are sometimes combined with other sensory inputs, such as haptic feedback, smells, and changing temperatures. Avatars (virtual characters with whom the user interacts) can be programmed to express emotions, for example, by blushing or crying. These high-fidelity avatars provide the user a greater sense of reality and facilitate meaningful interaction (2).

The field of virtual reality first came to light decades ago; however, recent advances in technology have made it the exciting and emerging field it is today. Its applications are vast, ranging from military training to gaming. In medicine, the technology has been trialed for uses such as cognitive rehabilitation post-stroke (3), improving reaction times in children with cerebral palsy (4) and in aiding the diagnosis of psychiatric conditions (5).

This paper sets out the viewpoint that virtual reality technology could be a new focus of direction in the development of training tools for medical education. We concentrate on its use in improving the communication skills of clinicians and medical students. We refer extensively to Pan et al.’s study, “The Responses of Medical General Practitioners to Unreasonable Patient Demand for Antibiotics—A Study of Medical Ethics Using Immersive Virtual Reality,” which explores the extent to which portable immersive virtual reality technology can help us gain an accurate understanding of the factors that influence a doctor’s response to an ethical dilemma. Pan et al. carried out a “proof of concept” research project, whereby twelve general practitioners (GPs) and nine GP trainees took part in a videoed 15-min virtual reality scenario. Participants were required to interact with two avatars: an elderly woman, the patient, and her daughter, who was requesting antibiotics for her mother’s likely viral infection. In essence, the doctor’s main goal was to try to resist calls for unnecessary antibiotics. The dilemma of tenacious calls for antibiotics by patients is a common yet difficult scenario due to the great threat posed by growing antimicrobial resistance worldwide. Responses of the avatar were pre-programmed, with a researcher selecting the most appropriate quote depending on what the participant said. The doctor undergoing the scenario was easily able to suspend reality owing to the highly immersive oculus rift headset. The videos were available after completing the session for reflection and to establish learning points.

Aside from exploring the potential of virtual reality technology as a training tool, the specific purposes of Pan et al.’s study were twofold: first, to investigate whether medical doctors would take the virtual situation seriously, and, second, whether experienced GPs would be more resistant to patient demands than GP trainees. A short video demonstrating the work can be seen at https://www.youtube.com/watch?v=C8Hs6NxtXB8. Results showed participants found it a useful and interesting experience. Experienced GPs were more able to say no to patients and uphold the principles of antibiotic stewardship, with trainees more likely to demonstrate poor prescribing behavior: eight of the nine trainees prescribed antibiotics, compared with seven of the twelve qualified GPs (1). Despite being only 15-min long, the scenario was very taxing and quite uncomfortable, as demonstrated by the facial expressions and body language of the participants and audience.

Pan et al.’s study focuses on the technical details of the virtual reality technology used, with some analysis of its success. However, the article was lacking in information regarding how useful the scenario was as a tool for medical education and antibiotic stewardship. We decided it was important to gather opinions and reflect further on how valuable the scenario was and the wider applications of virtual reality in medical education.

## Methodology

The content of this paper is a culmination of findings and feedback from the panel discussion at the Center for Behavior Change 3rd Annual Conference at University College London (UCL) on February 22, 2017. We held the panel discussion to consider how the short immersive virtual reality scenario might help change doctors’ prescribing behavior, what the limitations are and to discuss potential applications and implementations of virtual reality in medical education more broadly.

We selected a panel of experts from the following backgrounds: virtual reality, general practice, medicines management, medical education, and ethics. The aim of the panel was to invite questions and views from the audience and encourage balanced discussion of the issue from all of these angles. Attendees were asked to watch the video then each panel member (all of whom are authors) gave a brief dialog about one aspect of the issue:

(1) CS (general practitioner): antibiotic prescribing in general practice
(2) A-NL (pharmacist): antimicrobial stewardship
(3) CF (pediatrician): training tools in communication skills and the ethical considerations of virtual reality as a training tool
(4) XP (virtual reality lecturer): the technical details of the scenario with a focus on how avatars could be used in training
(5) SL (Clinical Leadership Fellow at NHS England): the benefits and potential negative implications for virtual reality as a training tool

The audience consisted of a range of healthcare professionals, commercial healthcare company representatives, public health professionals, behavioral science researchers, healthcare communication company representatives, and journalists. Unsurprisingly, there was a fair amount of dialog and disagreement as to the use of virtual reality in training medical professionals. The panel members reflected on the views offered during the panel discussion and compared them with their own. By culminating the attendees’ contributions with our thoughts, this article provides a critical analysis of the use of virtual reality in medical education from a variety of perspectives.

## Communication Skills in Medical Education

Communication skills training is now a core aspect of medical education, generally introduced early and continued throughout medical school. We believe that this is a skill that can be learned, practiced, honed and assessed. Many courses use the Cambridge-Calgary guide to the medical consultation as a useful framework for use in communication skills teaching (6). It can be broken down and its constituent parts used sequentially during both learning and examining. Medical students can modify the basic model depending on what they have observed in role models and what they feel most comfortable with. In this way, they are able to mold the consultation to ensure the patient’s satisfaction while remaining efficient.

Throughout training and in continuing professional development, medical students and doctors use roleplay with real and simulated patients to practice communication skills. One current training tool involves consultations with a patient actor followed by immediate feedback by experienced staff and the actor. They are often videoed and watched contemporaneously by a group, then kept for personal use to watch in a private space for deeper reflection and understanding. Another tool is to video real patient consultations with consent. This allows for both self-reflection and for an expert to analyze the consultation at a later time.

Communication skills training is also undertaken on the job, for example, in the form of work based assessments with senior colleagues or as part of a summative assessment for a specialist exit examination. Learning to communicate in complex or emotionally charged situations is challenging: it may occur by reflecting on things that could have gone better or, in worst case scenarios, when having to deal with a complaint, a claim or in court.

Often conflicts arise because we make assumptions about others we come into contact with, not least patients. It is important to reflexively consider how culture affects patient care. Aspects of culture such as race, religion, gender, sexuality, age, experience, background, language, and so on, undoubtedly affect the views medical professionals hold, and the way in which situations are handled. There are often stereotypes that must be abandoned while working with other professionals to be able to honestly and fruitfully work together. The use of virtual reality, where the appearance of avatars can be easily altered, may allow clinicians to gain a deeper understanding of their own values and how these affect their clinical practice.

Currently there is very little literature published comparing the use of virtual reality in consultation training compared with the current methods and this is something we plan to investigate in the future.

## Discussion and Emerging Themes

The workshop began by audience participants watching the avatar-doctor consultation video mentioned in the background section. They were then asked to reflect on their most recent experience of visiting a doctor for antibiotics and their impressions when the doctor decided to either give or refuse the prescription. The responses were wide-ranging and highlighted some of the various themes that can affect an individual’s expectations and approach to a consultation with a doctor.

First, geographical and cultural factors were found to influence an individual’s level of engagement and involvement in treatment decisions, and their attitudes toward antibiotics. A Danish participant commented that she was accustomed to doctors asking for her opinion on what treatment she needed, which included antibiotics (“what would you like?”). In comparison, German participants were more likely to expect the doctors to decide whether they needed antibiotics (“this is what I think you need”). People of different nationalities were generally aware that antibiotics must be used prudently.

Personality traits play a part in an individual’s approach to the consultation. Both the panel members and the audience remarked that some participants in the virtual reality scenario expressed apprehension in regards to having to decide on a treatment choice, preferring a paternalistic approach, while others would drive the consultation to the outcome that they desired. The outcomes of these consultations were felt to reflect the practitioner’s moral compass. Also coming into play were the practitioner’s own attitude toward potential confrontations, as well as their ability to persuade a patient (or family members) to consider a different approach to their problem.

The type of healthcare setting was thought to have an impact on an individual’s expectation of the consultation. Hospital attendances were perceived to be associated with more serious and complex diagnoses where treatment decisions would most likely be complicated, thus more guided by the clinician. Most participants responded that they would anticipate being told what to do and what is needed while in hospital (“in hospital I just do what I am told”). This was less so the case in general practice.

It was clear from the audience’s discussions that patients’ views surrounding antibiotic prescribing are wide-ranging and based on many different factors. In this respect, some people were skeptical that virtual reality technology would be able to reflect the diversity and complexity of patients’ responses to discussions regarding antibiotics.

## Viewpoint

Changing clinicians’ behavior concerning decision-making is complicated. Understanding the clinical and social complexity and recognizing the inter-personal factors involved is key. The principles of being self-aware and reflective are undeniably essential. The decision to prescribe antibiotics is often multifactorial, with GPs having to take a number of factors into account besides the patient’s presenting complaint. The matter of antibiotic prescribing has been addressed comprehensively in recent years. From practice and personal audits, to “carrot and stick” approaches by Medicine Management Teams at Clinical Commissioning Group level––much effort and funding has been put in place to try to understand and tackle the continuing issue of antibiotic overprescribing. Research into the use of antibiotics often focuses on collecting data about the current situation, and the consequences of overuse, however simply recognizing it is a problem that has limited impact in changing behavior.

In our opinion, studying antibiotic overuse using virtual reality technology gives us a better understanding into why the problem persists. This allows more effective and personalized education on the subject. It is a common scenario with potentially huge implications on the future of public health, making it an important focus of medical education. Research conducted into prescribing behavior and subsequent protocols and guidelines often focus on the clinical condition, its natural course, and side effects of antibiotic treatments. Virtual reality consultations bring a different perspective to the traditional way in which prescribing has been researched; it incorporates the patient’s (avatar’s) interaction and allows the clinicians’ responses to be studied, not only by an audience but also by the clinician themselves. One audience member described how virtual reality allows you to “recognize the emotions you go through as part of a consultation.”

Reactions to the doctor-avatar consultation were mixed. Many recognized their own experiences of similar consultations. They discussed feelings of empathy toward both the doctor and avatar, expectations of “right or wrong” practice, uncertainty and an overall feeling of discomfort.

The virtual reality scenario highlighted the complex nature of decision-making and demonstrated to the audience that the clinician was making a decision based not just on clinical evidence or the latest national or local guidelines. It also exposed the uncomfortable conflict that arises within the doctor’s mind during their thought process, knowing that they may cause distress or lose the patient’s trust should they refuse antibiotics. Doctors were being asked to take an empathic approach to the patient’s unique situation and decide the management appropriately. The doctor may try one approach in a virtual reality consultation then view their performance and listen to advice on other approaches that could benefit their skills. The doctor can replay the same scenario and see how different approaches, explanations and reactions change the consultation.

There are many situations in which virtual reality consultations may be preferable to the use of patient actors. In pediatrics, it is ethically inappropriate to use child actors as victims of abuse for the benefit of teaching. However, practicing child protection scenarios is vital in educating clinicians to notice signs of abuse and react appropriately. Furthermore, consultations with child actors, particularly under the age of five, are particularly challenging due to the unpredictability of child actors. Virtual reality may provide a solution to these respective concerns.

Our view is that virtual reality provides a safe environment to learn and reflect. Coming across aggressive patients or family members is an unfortunate eventuality in a doctor’s career, with conditions such as dementia and mental illness sometimes playing a part. Virtual reality technology was thought to be particularly useful in such situations in order to protect the doctor’s safety. It allows clinicians to take a “trial and error” approach to learning how to respond to these situations, rather than going through this process and putting themselves in danger in real life.

Virtual reality may allow the user to work remotely, facilitating distance learning. Clinicians could carry out scenarios at home, providing a novel way to practice communication skills without the need for real actors.

On the other hand, there are various downsides and barriers to the use of virtual reality in medical training. In its current form, avatars are not especially realistic and this may make clinicians less able to immerse themselves in the scenario. Doctors may feel more able to say no to an avatar than a real person. With added funding, the technology could become more sophisticated, with speech recognition and more realistic facial expressions. Creating higher fidelity avatars requires greater funds: another barrier to implementation.

The scenarios have so far only allowed observation of behavior, not demonstration of behavior change. Clinicians may have acted differently knowing they were taking part in research rather than communication skills training, making the results potentially less valid. More research is needed to compare the effectiveness of teaching using virtual reality against using role players and to further explore barriers to implementation.

## Conclusion

In conclusion, virtual reality was found to be a useful training tool, one that may succeed in cases where other initiatives have failed to induce behavior change. Through the virtual reality consultation, a doctor can develop greater self-awareness and modify their future reactions in a better therapeutic way. While antibiotic prescribing is a useful example, the use of patient-avatars is applicable to many scenarios. With greater funding and improved technology, there is clearly scope for higher fidelity scenarios. However, there are certain limitations to the study, namely that more data is needed to demonstrate behavior change in comparison to other training modes, the high costs involved, and that the technology was not trialed in a “real life” training environment.

## Author Contributions

CF facilitated the workshop and the writing up of this research. PA-W compiled sections and wrote up the research. CS, A-NL, and SL were at the workshop and provided viewpoints and backgrounds. SD was the instigator of the original project. XP undertook the virtual reality, was present at the workshop, and provided viewpoints and background.

## Conflict of Interest Statement

The authors declare that the research was conducted in the absence of any commercial or financial relationships that could be construed as a potential conflict of interest.
